# Grass pollen production and group V allergen content of agriculturally relevant species and cultivars

**DOI:** 10.1371/journal.pone.0193958

**Published:** 2018-03-12

**Authors:** Stephan Jung, Nicole Estrella, Michael W. Pfaffl, Stephan Hartmann, Ellinor Handelshauser, Annette Menzel

**Affiliations:** 1 Department of Ecology and Ecosystem Management, Technical University of Munich, Freising, Germany; 2 Department of Animal Physiology & Immunology, Technical University of Munich, Freising, Germany; 3 Institute for Plant Production and Plant Breeding, Bayerische Landesanstalt für Landwirtschaft, Freising, Germany; 4 Institute of Advanced Study, Technical University of Munich, Garching, Germany; Chang Gung University, TAIWAN

## Abstract

Grass pollen is the main cause of hay fever and allergic asthma in warm temperate climates during summer. The aim of this study was to determine the content of group 5 major allergens in pollen grains of agriculturally important grass species/cultivars. For each cultivar flowering dates and pollen production of cut anthers were observed in the field and in a climate chamber, respectively. An ELISA was used to quantify the group 5 allergens (Phl p5) in pollen extracts which were gained from the grass species Kentucky bluegrass, perennial rye grass, timothy, cocksfoot, annual / Italian rye grass, hybrid rye grass and festulolium. The group 5 allergen content of species varied between 0.01 ng (Kentucky bluegrass) and 0.06 ng (timothy) per pollen grain. On cultivar level the pollen allergenic content differed up to 74-times within the selected grass species. Results from this study might be helpful for the reduction of allergen exposure coming from agriculture grass production e.g. by an adapted grass selection or by the cultivation of grasses with low allergenic content in plant breeding.

## Introduction

Grass pollen is, besides birch pollen, one of the most frequent reasons for plant related allergenic reactions worldwide [1]. People who are sensitive or allergic to pollen develop symptoms such as hay fever, allergic rhinitis or even allergic asthma. At present 13 different groups of allergens related to grass pollen are known which induce IgE responses [2], of which 11 groups can be found in *Pooideae* pollen, a subfamily of the *Gramineae* or *Poaceae* family [3]. Among these allergens, group 1 (glycoproteins, molecular mass of 32–35 kDa) and group 5 allergens (glycoproteins molecular mass 28–32 kDa) are the major allergens, since they induce allergenic reactions in patients at high rates (65–85% of patients allergic to grass pollen are sensitive to group 5 and 90–95% to group 1 allergens) [3–5]. Grass pollen (~35–65 μm in diameter) itself is not tiny enough to enter lower airways and thus to directly cause respiratory allergies. Rather, when pollen is dissolved its allergenic content is released in form of small granules (0.6–2.5 μm). These granules can bind to smaller particles, e.g. fine dust, and thus are small enough to cause allergenic reactions, also in the distal parts of the lungs [6].

Worldwide there are 9,000 different grass species (*Poaceae*) [2], nevertheless in German agriculture only about 20 of them achieve higher coverage in the area. But in fact one species may comprise lots of varieties. For example, about 300 different cultivars of perennial rye grass (*Lolium perenne* L.) are listed in Germany. Cultivars have been developed by plant breeders for specific purposes, such as maximizing agricultural yield under certain site conditions or ornamental aims. Thus, pollen allergenicity of different cultivars has been—to our knowledge completely—neglected due to its limited relevance in agriculture and horticulture. Limited data only exists on the species level (e.g. [7]).

In general, pollen concentrations in the air are highly dependent on the timing and duration of flowering as well as short- and long-range atmospheric transport and weather conditions. For grass, agricultural management is another impact factor, since hay cutting or in more recent time’s silage cutting might take place before most grass species reach the flowering phase, resulting in a considerable smaller pollen release. Warmer late winter / early spring conditions due to anthropogenic induced climate change advance the spring phenology of many plant species in the temperate and boreal zone [8], therefore also grass species have shown a trend towards earlier flowering in the recent decades [9]. For Germany, most interestingly, a divergence between flowering and hay cutting dates has been revealed [10]. More specifically, flowering dates tended to be earlier whereas hay cutting dates did not advance during the last decades. This phenomenon can be explained by management decisions among others historically fixed hay cutting dates or subsidies [11] in Agri-Environmental schemes for extensification (later first silage cutting leading to reduced number of cuts per year or land set-aside) to e.g. preserve biodiversity [12]. If farmers’ annual activities are not tracking the speed of climate change in their grassland management [13], this divergence would allow at the end more grass species to come into flowering and consequently more allergenic pollen to be released into the atmosphere. On the other hand, due to the fact that hay production was largely replaced by silage production and thus there are an increased number of cuts per year in the past decades, the first silage cut should be antedated. The number of cuts has a significant influence on the annual agricultural income [14]. Additionally, from the agricultural perspective it is very important that the silage cut should take place when ear/panicle emergence of the dominant species in the sward (pointed foxtail *Alopecurus pratensis*) occurs as the fodder quality immediately decreases after flowering [15,16]. Potential adaptations in agriculture management towards climate change from farmers side depend on their awareness [17]. Anyhow, strategies for a sustainable intensification in management are already discussed [18].

Since the number of persons allergic to grass pollen is high and still increasing, more effort should be put into the investigation of the allergen content in species and cultivars in order to provide improved allergen-specific recommendations in the selection and cultivation of cultivars, not only in agriculture, but especially in landscape building, gardening, and urban landscaping as these cultivation forms are exclusively driven by seedlings. Studies on mean allergen exposure mostly rely on daily pollen concentrations/counts derived from volumetric pollen samplers. At present an optical identification and distinction of pollen from different grass species is not possible [19], although most grass species do not cause allergies at all. Therefore grass pollen counts only to a certain degree mirror the related allergenic potential. Furthermore, the allergenic potential strongly depends on weather conditions, season of the year and geographical location making it difficult to predict allergen exposure only by pollen concentration [20].

Therefore, it is important to know to which degree pollen of the selected grass cultivars vary in their amount and allergen content, and whether allergen content and pollen production are related to flowering dates of the cultivars.

To address this question we chose 15 different grass cultivars due to the allergenicity of their pollen [21], their prevalence in seed mixtures, different heading date, ploidy and genetic background. We collected grass shortly before flowering on a grass cultivar trial field in 2016 and analyzed the amount of pollen produced, and group 5 allergenic content of the pollen.

The amount of major group 5 allergens on species/cultivar level was determined by molecular techniques [22], and pollen productivity was analyzed by pollen count. Phenology and particular flowering dates were recorded according to the BBCH code [23]. Results show that there are large differences in allergenic content between the selected cultivars. They may help to optimize the choice of grass species, to revise agricultural and landscape management practices and/or support plant breeders in their efforts to breed cultivars with down-regulated allergens [24].

## Material and methods

### Investigated grass species and cultivars

The following seven grass species were analyzed for their allergenic content: Kentucky bluegrass (*Poa pratensis* L.), perennial rye grass (*Lolium perenne* L.), timothy (*Phleum pratense* L.), orchard grass (*Dactylis glomerata* L.*)*, annual / Italian rye grass (*Lolium multiflorum* Lam.), hybrid rye grass (*Lolium x hybridum)*, and festulolium (*Festuca spec*. *x Lolium spec*.). In total 15 cultivars were chosen for this study, one annual and one hybrid rye grass, two cultivars each of Kentucky bluegrass, orchard grass, timothy and hybrid fescue as well as five cultivars of perennial rye grass (see Table 1 for a complete list of the cultivars). All grass cultivars used in this study passed Distinctness, Uniformity and Stability (DUS) procedures, thus it is guaranteed, that varieties are authentic variants from the respective species. The seeds of the cultivars planted by Bavarian State Research Center for Agriculture (LfL) have the quality level of seeds used in Value or Cultivation and Use (VCU)-trials. So seeds of high defined genetic standard were ordered directly from the breeder or the responsible maintainer of the respective variety. The cultivars selected vary mainly in their flowering time.

### Experimental site

The experimental field site was located at 450 m a.s.l. in Pulling (southern part of Germany, close to Munich / Freising, 48.3712°N, 11.7181°E) and is operated by the LfL. This trail field site exists since 2014 and consists of various grass species and cultivars which are grown for mainly educational purposes. Each cultivar is planted on a 1.5 m x 7 m subplot.

### Phenological, meteorological and airborne pollen data

From beginning of May till end of June 2016 vegetative and reproductive phenological stages of all cultivars (except for festulolium and annual rye grass cultivars, which were included later in the study) were recorded with the expanded BBCH code on a weekly basis following the description of Meier [23]. Observations included the macro stage 4 (booting), 5 (inflorescence emergence, heading), 6 (flowering, anthesis), 7 (development of fruit), 8 (ripening) to macro stage 9 (senescence). The mean weekly (micro-) stage was derived by averaging the (micro-) stages recorded for each of the subplots. Linear interpolation was used to receive the exact flowering dates (BBCH 61).

Airborne pollen concentration was measured during the vegetation period of 2016 using a 7 Day Recording Volumetric Spore Sampler (Burkard Scientific Ltd, Uxbridge, UK) which was installed in 15m height above ground at the forest faculty building of the Technical University of Munich in Freising, Germany (48.3999°N, 11.7180°E) and was there attached to the meteorological platform at the east side of the building. The air flow was set to 10 L/min. Pollen grains were trapped on an adhesive tape which was fixed on a drum and driven by a clockwork motor. After recording, the sampling tape was cut into half day sections which were than preserved on microscope slides. Grass pollen were identified and counted by light microscopy following the requirements for pollen monitoring [25].

Meteorological data (air temperature, air humidity and precipitation) in hourly resolution were obtained from a nearby climate station (Weihenstephan-Dürnast, location 48.4029°N; 11.7305°E, distance to trial field site 3.5 km and distance to meteorological platform 1.0 km) of the German Meteorological Service (DWD) and used for characterizing the meteorological growing conditions.

### Collection of grass pollen and pollen count per culm

When first flowering (BBCH code 61) was observed for a cultivar, bunches of 50–60 individual plants were harvested randomly from the plot and inflorescences were covered with pergamin bags. Pollen collection directly on the field turned out to be impossible due to adverse environmental conditions (rain, herbivore insects). After attaining the full flowering stage (BBCH code 65) under fixed conditions in a climate chamber (day/night cycle: 14 hours day at 23°C, 10 hours night at 15°C; air humidity 45%), pollen was extracted from the pergamin bags by shaking and subsequent removal of anthers and culms. Additionally the number of culms per bag was counted and the total pollen weight was determined by an electronic balance (Mettler Toledo, model XS204DR). This method for pollen collection and isolation similar to [26] was consistently applied for all samples. However we cannot exclude that single pollen still adhered to the bags or were not released by the anthers at that time.

### Extraction and determination of protein content

100 mg pollen grains were mechanically decomposed by shaking pollen continuously at room temperature for three hours in PBS (phosphate buffered saline) [27,28]. Afterwards the dispersion was centrifuged at 13.600 rpm for 5 min. Analysis of total soluble protein content was conducted with a classical BCA test [27,28]. BCA solution and copper sulfate were obtained from Sigma (B9643; C2284). For the standard curve Albumin from Serva (11930) was used and its range was 25–1000 μg/ml concentration.

### Grass pollen weight and allergen quantification

5 mg pollen grains of each grass cultivar sample were weighed, dissolved in 250 μl PBS and then the amount of pollen were counted (n = 4) by an automated cell counter (TC-10, Bio-Rad Laboratories GmbH, München, DEU) [28]. Results from these four counts were then averaged. Group 5 allergen quantification was carried out by a commercial available sandwich ELISA (Allergopharma GmbH, Reinbeck/Hamburg, Germany) [7,22,29]. Within this test the timothy (*Phleum pretense*) group 5 allergen Phl p5 served as reference [30] and the monoclonal antibodies MoAb 1D11 and MoAb B01 (Allergopharma GmbH) were used to detect the available epitopes present on the grass pollen allergens Phl p5a and Phl p5b. The biotinylated second antibody B01 together with a chromogen generated spectrophotometrically detectable signals visualizing the epitope-antibody binding. Based on the standard material a standard curve from 1 to 1000 ng/ml concentration was created. The allergen quantification of all group 5 allergen by ELISA allowed a sensitivity of 1ng/ml and showed a precision of ±10% [31]. The antibodies MoAb 1D11 and MoAb B01 can be used for the quantification of group 5 allergen content in soluble extracts from all species of *Poaceae* as group V allergens are homologous proteins in grass pollen [32,33].

### Statistics

The data were analyzed with R [34] using the packages ggplot2, latticeExtra, tidyr and ggpubr. Data were tested for normal distribution using the Shapiro test. Correlation analysis for nonparametric data was calculated by Spearman’s Rank Correlation. P values smaller than 0.05 were considered as statistically significant.

## Results

### Airborne pollen counts and weather in spring 2016

**Fig 1:**
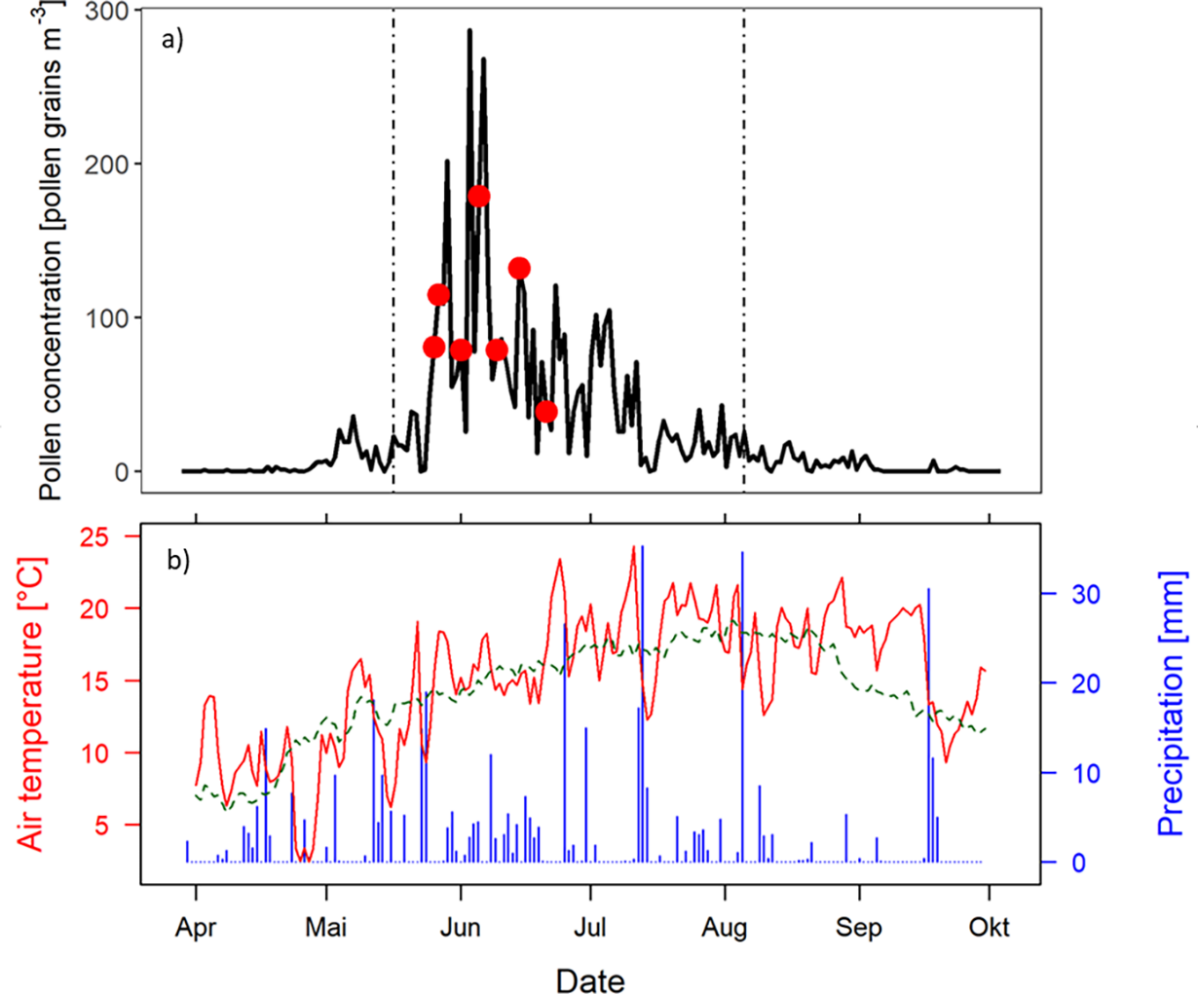
a) Pollen concentration (pollen grains m^-3^). Dashed lines indicate thresholds when 5 and 95% of grass pollen are released. Red points represent start of flowering for the investigated cultivars b) Air temperature (°C) and precipitation (mm) from March 3^rd^ to October 15^th^ 2016. Green dashed line shows averaged air temperature between 1987 and 2016.

In the sampling period (03.03. - 15.10.2016), an average daily concentration of 21 grass pollen m^-3^ and a total amount of 4,800 pollen m^-3^ for the whole vegetation period were measured. Start and end of grass pollen season (5% and 95% of year’s total amount reached) were on 18.05.2016 and 04.08.2016. Highest grass pollen concentrations with up to 268 pollen m^-3^ per day (04.06.2017) were reached between end of May and mid of June (see Fig 1). Mean air temperature in the sampling period was 13.3°C, and precipitation was around 500 mm in total. In comparison to the average climate conditions from 1987 to 2016, air temperature in 2016 was around 0.5°C higher and total precipitation was nearly 60 mm lower. For April, May and June average monthly air temperatures of 8.4°C, 12.9°C and 16.6°C were measured. The precipitation was 49 mm in April in comparison to Mai and June (100 mm and 105 mm) (Fig 1).

### Phenology

**Fig 2:**
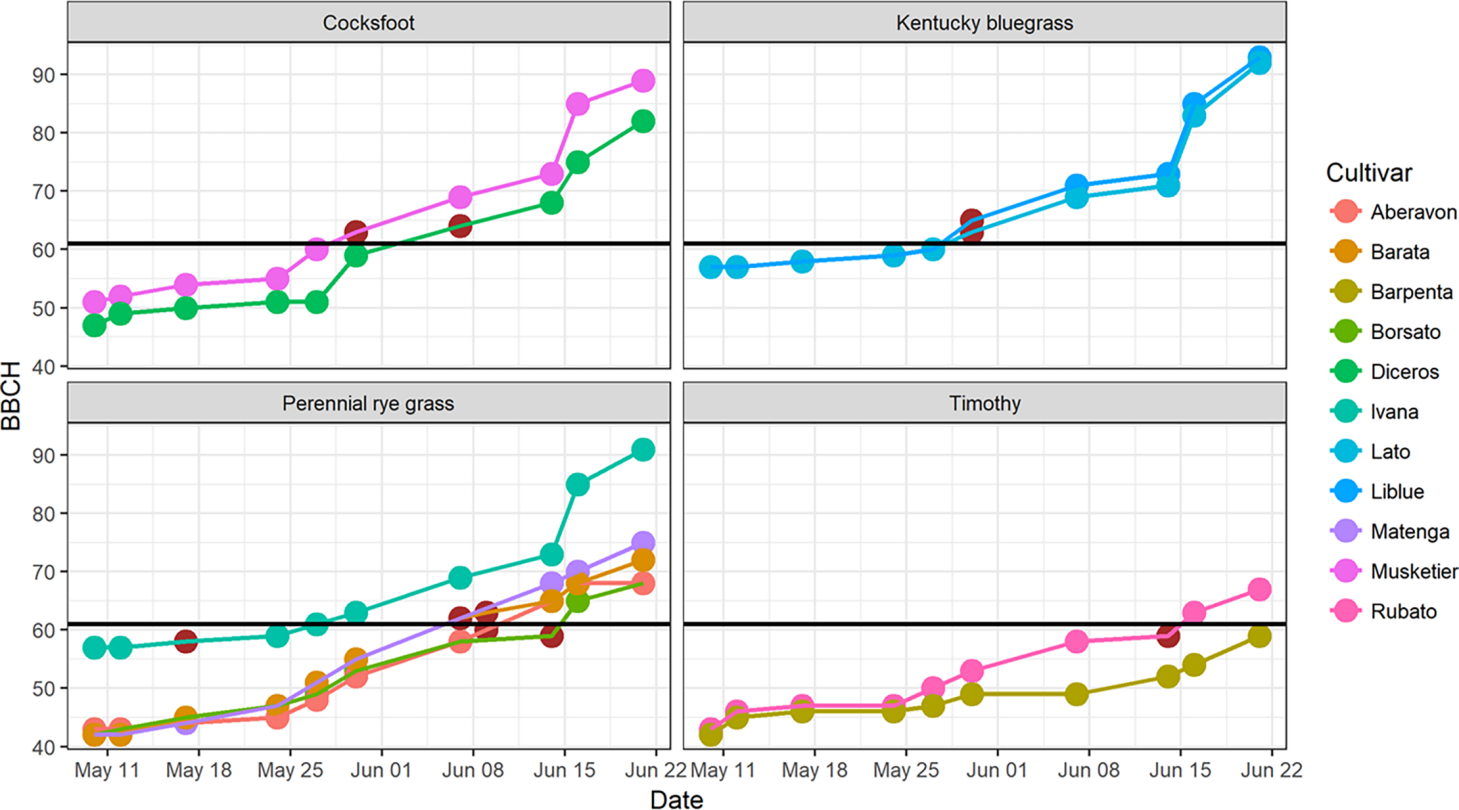
Phenological development of different grass species and cultivars in spring 2016. BBCH indicates the (micro-) stages according to BBCH code [23] between May 10^th^ and June 21^st^ 2016. Black line indicates start of flowering (BBCH 61). Dark brown points represent the date of harvesting on the field for analysis of allergen content.

Among the selected cultivars, Ivana (perennial rye grass cultivar), Liblue and Lato (Kentucky bluegrass cultivars) and Musketier (orchard grass cultivar) were the first to start flowering (BBCH code 61) on May 27 and 28 (day of the year (DOY) 148 and 149) (Fig 2). The first flowering for Diceros (cocksfoot cultivar) was determined on DOY 154. For Matenga, Barata and Aberavon (perennial rye grass cultivars) flowering was observed between DOY 158 and 162 (middle to late flowering cultivars). Borsato (perennial rye grass cultivar) and Rubato (timothy cultivar) started flowering at DOY 162 and 167 (late). Barpenta (timothy cultivar) flowered latest on June 21 (DOY 173). Independent from cultivar, Kentucky bluegrass and cocksfoot flowered early and timothy was a late flowering species whereas within perennial rye grass there was a large range of flowering dates of the different cultivars. Since we only have data from one season and one site, thorough statistical analyses are not possible. The first peak in atmospheric grass pollen concentrations matched with the start of flowering of the earliest cultivars, whereas first flowering of the latest cultivars was within the smaller peaks towards the end of the grass pollen season (see Fig 1).

Harvesting of plant material for pollen collection in the climate chamber took place shortly after first flowering (BBCH 61) was observed for the early flowering species Kentucky bluegrass (2 cultivars) and cocksfoot (2) as well as for 2 out of 5 cultivars of perennial rye grass. In contrast, for the late flowering species timothy (2 cultivars) as well as for 2 late flowering cultivars of perennial rye grass the harvesting was done shortly before first flowering. Only Ivana (perennial rye grass) was harvested considerably earlier than the first flowering date.

### Pollen allergen content

For the investigated cultivars the mean observed pollen grain weight was 20.2 ng (Tab. 1) whereas the highest mass (41.7 ng grain^-1^) was reached by Hera (Italian rye grass cultivar) and the lowest mass (7.5 ng grain^-1^) was found for Lato (Kentucky bluegrass cultivar). These values are matching with total protein content, for which Hera had the highest value (5.9 ng grain^-1^) and Lato again the lowest one (0.9 ng grain^-1^), respectively. In average, pollen extracts had a total protein content of 11.64 μg / ml which is in good agreement to other studies [27]. The mean total protein amount per pollen grain was 2.5 ng.

In the extracts from different cultivars there was on average 138 μg/ml group 5 allergen concentration, ranging—on the species level—from 90 μg/ml (perennial rye grass) to 295 μg/ml (timothy). The lowest allergen content among all cultivars was found in Abervaron (perennial rye grass cultivar) with 36 μg/ml and the highest in Barpenta (timothy cultivar) with 324 μg/ml (Table 1).

**Table 1:** Allergen content of 15 allergenic grass cultivars. Soluble group 5 allergens in solution; mass per pollen grain, soluble protein and group 5 allergens per pollen grain; percentage of soluble protein and group 5 allergens per total grain mass and percentage of soluble group 5 allergens per total soluble protein in pollen extracts; NA = not available (too little of pollen).

Grass species	Cultivar	Soluble group 5 allergen content (μg/ml)	Mass per pollen grain (ng/grain)	Soluble protein per pollen grain (ng/grain)	Soluble group 5 allergen per pollen grain (ng/grain)	Soluble protein per total grain mass (%)	Soluble group 5 allergen per total grain mass (%)	Soluble group 5 allergen per total soluble protein (%)
Kentucky bluegrass	WR Lato	123.42	7.49	0.86	0.009	11.43	0.12	1.08
Kentucky bluegrass	WR Liblue	113.24	12.27	1.52	0.014	12.36	0.11	0.92
**Average Kentucky bluegrass**		**118.33**	**9.88**	**1.19**	**0.012**	**11.89**	**0.12**	**1.00**
Festulolium	FL Lesana	163.65	19.34	2.75	0.032	14.22	0.16	1.15
Festulolium	FL Paulita	122.51	NA	NA	NA	NA	NA	NA
**Average Festulolium**		**143.08**						
Hybrid rye grass	BW Pirol	113.52	NA	NA	NA	NA	NA	NA
Italian rye grass	WW Hera	114.86	41.67	5.85	0.048	14.05	0.11	0.82
Cocksfoot	KL Diceros	186.28	16.67	2.11	0.031	12.64	0.19	1.47
Cocksfoot	KL Musketier	89.44	23.95	3.36	0.021	14.04	0.09	0.64
**Average Cocksfoot**		**137.86**	**20.31**	**2.73**	**0.026**	**13.34**	**0.14**	**1.06**
Perennial rye grass	WD Aberavon	36.31	12.50	1.51	0.005	12.08	0.04	0.30
Perennial rye grass	WD Barata	99.38	12.50	1.47	0.012	11.76	0.10	0.85
Perennial rye grass	WD Borsato	129.79	21.76	2.23	0.028	10.26	0.13	1.27
Perennial rye grass	WD Ivana	96.59	26.53	2.12	0.026	8.00	0.10	1.21
Perennial rye grass	WD Matenga	90.70	27.89	3.21	0.025	11.51	0.09	0.79
**Average Perennial rye grasses**	** **	**90.55**	**20.24**	**2.11**	**0.019**	**10.72**	**0.09**	**0.88**
Timothy	WL Barpenta	324.39	16.39	2.01	0.053	12.27	0.32	2.64
Timothy	WL Rubato	265.70	23.67	3.06	0.063	12.93	0.27	2.06
**Average Timothy**		**295.04**	**20.03**	**2.54**	**0.058**	**12.60**	**0.30**	**2.35**
**Overall Average**		**137.98**	**20.20**	**2.47**	**0.028**	**12.12**	**0.14**	**1.17**

On the pollen grain level an average allergen content of 0.03 ng was observed. In relation to grain mass, the group 5 allergen proportion was 0.14%, ranging—on the species level—from 0.09% (perennial rye grass cultivar) to 0.30% (timothy cultivar). On cultivar level the smallest proportion was found in Aberavon (0.04%, perennial rye grass cultivar) and highest proportion was encountered in Barpenta (0.32%, timothy cultivar). Compared to other cultivars, Barpenta was growing in clusters poorly covering ground, and flowering was observed six days after the other cultivars (Fig 2). Allergen proportions correlated reasonably well with flowering dates of cultivars (r = 0.46, n = 11, p = 0.15, not significant), indicating for the later flowering cultivars higher allergen proportions.

On average 5,400 ng group 5 allergen was observed per culm whereby the highest amount was reached by Rubato (14,200 ng, timothy cultivar) and the lowest in Aberavon (192 ng, perennial rye grass cultivar). Thus, Rubato has a 74-times higher group 5 allergen content at culm level in comparison to Aberavon.

## Discussion

The group 5 allergen (Phl p5) is one of the largest triggers of hay fever and allergic asthma. Phl p5 is a protein with two different isoforms and high variability of IgE epitopes explaining its high allergenic potential [35]. It can be found exclusively in the grass Pooideae subfamily [36,37]. The determination of allergen content focused on the group 5 as it is well detectable in various grass species using the ELISA technique [7] whereas allergen quantification in other allergen groups is known to be not accurate (group 1 allergens) or allergen potential is low. Nevertheless other allergen groups (especially group 1) are important causes for the high allergenic potential of pollen as well. In general, group 5 allergens are used in specific immunotherapy as a reference for allergen standardization [38]. This study provides an overview on the group 5 allergenic potential for the most important grass species / cultivars used in agriculture. For the first time a comparison between different grass cultivars and their content of major grass group 5 allergen was performed. In general, our phenological surveys and the measurement of atmospheric grass pollen concentrations indicated that the period between end of May to end of June is most problematic for people allergic to grass pollen.

The allergen content was measured by a quantitative ELISA analysis of pollen extracts which were gained from field samples. As pollen collection directly outside in the field is quite challenging and with limited success due to adverse environmental conditions, it was only possible to determine the allergenic content once during flowering period and in an assist climate chamber.

On average 0.03 ng of group 5 allergens per grass pollen grain was observed which is in good agreement with a comparable study [7] where 0.05 ng per grass pollen grain was found. The allergen content in our study varied up to 74-times within the selected cultivars demonstrating the high variability of their allergenic potential. We observed a mean mass per pollen grain of 20.20 ng whereas [7] found 11.0 ng. The average percentage of soluble protein was 12.1%. Other studies found 2%, 6% [7] and 8% [27]. Those large differences in protein content might be explained by the fact that the determination of the soluble protein content is highly dependent on the standard used in the test and thus can vary between studies.

Based on the phenological recordings carried out using BBCH code [23], the date of first flowering varied up to 25 days between cultivars, nevertheless all observed flowering dates were in well coincidence with reference values provided by the German Bundessortenamt [39]. There was no significant correlation between flowering date and cultivar-specific allergen proportions.

Provided that the detected allergenic content remains constant during the flowering period, cultivars from the species Kentucky bluegrass, italian rye grass and perennial rye grass should be grown preferentially in respect to minimizing the allergen production. Since variability in allergenic content for perennial rye grass is quite high, more cultivars from this species have to be investigated to provide solid recommendations. Due to the high allergenic content and pollen production for both investigated timothy cultivars, the cultivation of especially this species should be kept to a minimum level.

Nevertheless it is questionable whether cultivars from this species grown on intensively cultivated areas may reach flowering at all before harvesting since plant development is quite slow and thus flowering is late in comparison to most of the other species. In intensive grassland management, only a few early flowering cultivars / species, which are commonly used as indicators for sward development, come into flowering before the first silage cut, since protein content of the biomass sharply decreases when grass species flower. Consequently the local (potential) emission of grass allergens [10] and thus actual strengths of hay fever symptoms may be influenced by the time of hay cutting.

The genus bastard hybrid fescue (rye grass x meadow fescue cultivar) has on average higher soluble group 5 allergen content compared to perennial rye grass. This would mean that the allergenic content of perennial rye grass rises during the hybridization with fescue.

As grass pollen allergens are a strong trigger for hay fever and asthma [40] investigations about the allergenic potential of grasses extensively used in agriculture are absolutely necessary. Future studies are needed to analyze whether there are cultivar-specific peaks in the allergen content during plant development and thus during the grass pollen season, and to which extent meteorological conditions or respectively climate change have an impact on the allergen content.

## Conclusion

The analysis of the group 5 content in different grass species/cultivars offers new insights into the allergenic risk from grasses (*Poaceae*) for patients suffering from hay fever. The study revealed that Kentucky bluegrass, italian rye grass and perennial rye grass have the least allergenic content and thus should be grown preferentially whereas timothy should be kept to a minimum.

## Supporting information

S1 Table (XLSX)

## References

[1] D'Amato G, Cecchi L, Bonini S, Nunes C, Annesi-Maesano I, Behrendt H, et al Allergenic pollen and pollen allergy in Europe. Allergy. 2007; 62: 976–990. doi: 10.1111/j.1398-9995.2007.01393.x 17521313 10.1111/j.1398-9995.2007.01393.x

[2] Andersson K, Lidholm J. Characteristics and immunobiology of grass pollen allergens. Int Arch Allergy Immunol. 2003; 130: 87–107. doi: 10.1159/000069013 12673063 10.1159/000069013

[3] Hrabina M, Peltre G, van Ree R, Moingeon P. Grass pollen allergens. Clinical & Experimental Allergy Reviews. 2008; 8: 7–11. doi: 10.1111/j.1472-9733.2008.00126.x

[4] Duffort O, Quintana J, Ipsen H, Barber D, Polo F. Antigenic similarity among group 1 allergens from grasses and quantitation ELISA using monoclonal antibodies to Phl p 1. Int Arch Allergy Immunol. 2008; 145: 283–290. doi: 10.1159/000110887 18004069 10.1159/000110887

[5] Marcucci F, Sensi L, Incorvaia C, Dell'Albani I, Di Cara G, Frati F. Specific IgE response to different grass pollen allergen components in children undergoing sublingual immunotherapy. Clin Mol Allergy. 2012; 10: 7 doi: 10.1186/1476-7961-10-7 22694773 10.1186/1476-7961-10-7 PMC3511885

[6] Suphioglu C, Singh MB, Taylor P, Knox RB, Bellomo R, Holmes P, et al Mechanism of grass-pollen-induced asthma. The Lancet. 1992; 339: 569–572. doi: 10.1016/0140-6736(92)90864-Y 10.1016/0140-6736(92)90864-y 1347092

[7] Schappi GF, Taylor PE, Pain MC, Cameron PA, Dent AW, Staff IA, et al Concentrations of major grass group 5 allergens in pollen grains and atmospheric particles: implications for hay fever and allergic asthma sufferers sensitized to grass pollen allergens. Clin Exp Allergy. 1999; 29: 633–641. 10231323 10.1046/j.1365-2222.1999.00567.x

[8] Rosenzweig C, Karoly D, Vicarelli M, Neofotis P, Wu Q, Casassa G, et al Attributing physical and biological impacts to anthropogenic climate change. Nature. 2008; 453: 353–357. doi: 10.1038/nature06937 18480817 10.1038/nature06937

[9] Munson SM LA. Climate drives shifts in grass reproductive phenology across the western USA. New Phytol. 2017; 213: 1945–1955. doi: 10.1111/nph.14327 27870060 10.1111/nph.14327

[10] Bock A, Sparks TH, Estrella N, Menzel A. Changes in the timing of hay cutting in Germany do not keep pace with climate warming. Glob Chang Biol. 2013; 19: 3123–3132. doi: 10.1111/gcb.12280 23744623 10.1111/gcb.12280

[11] Klimek S, Richter KA, Steinmann HH, Freese J, Isselstein J. Rewarding farmers for delivering vascular plant diversity in managed grasslands. A transdisciplinary case-study approach. Biological Conservation. 2008; 141: 2888–2897. doi: 10.1016/j.biocon.2008.08.025

[12] Klimek S, Marini L, Hofmann M, Isselstein J. Additive partitioning of plant diversity with respect to grassland management regime, fertilisation and abiotic factors. Basic and Applied Ecology. 2008; 9: 626–634. doi: 10.1016/j.baae.2007.11.005

[13] Menzel A, Vopelius J, Estrella N, Schleip C, Dose V. Farmers' annual activities are not tracking the speed of climate change. Clim Res. 2006; 32: 201–207. doi: 10.3354/cr032201

[14] Petersen-Schlapkohl T, Isselstein U, Isselstein J. Produktivität und Futterqualität von naturnahem Grünland unterschiedlicher funktioneller Diversität unter verschiedener Bewirtschaftungsintensität; 2011.

[15] DLG-Futterwerttabellen Wiederkäuer. 8th ed. Frankfurt am Main: DLG-Verlag; 2007.

[16] Brum OB, López S, García R, Andrés S, Calleja A. Influence of harvest season, cutting frequency and nitrogen fertilization of mountain meadows on yield, floristic composition and protein content of herbage. R. Bras. Zootec. 2009; 38: 596–604. doi: 10.1590/S1516-35982009000400002

[17] Eggers M, Kayser M, Isselstein J. Grassland farmers’ attitudes toward climate change in the North German Plain. Reg Environ Change. 2015; 15: 607–617. doi: 10.1007/s10113-014-0672-2

[18] Volkmar W, Isselstein J, Stützel H, Ordon F, Haaren C, Schlecht E et al Nachhaltige ressourceneffiziente Erhöhung der Flächenproduktivität: Zukunftsoptionen der deutschen Agrarökosystemforschung Grundsatzpapier der DFG Senatskommission für Agrarökosystemforschung. 586 KB / Journal für Kulturpflanzen 66(7) 2014 2014. doi: 10.5073/JFK.2014.07.01

[19] Jaeger S. Exposure to grass pollen in Europe. Clinical & Experimental Allergy Reviews. 2008; 8: 2–6. doi: 10.1111/j.1472-9733.2008.00125.x

[20] Buters J, Prank M, Sofiev M, Pusch G, Albertini R, Annesi-Maesano I, et al Variation of the group 5 grass pollen allergen content of airborne pollen in relation to geographic location and time in season. J Allergy Clin Immunol. 2015; 136: 87–95.e6. doi: 10.1016/j.jaci.2015.01.049 25956508 10.1016/j.jaci.2015.01.049

[21] Ramirez J, Obispo TM, Duffort D, Carpizo JA, Chamorro MJ, Barber D, et al Group 5 determination in Pooideae grass pollen extracts by monoclonal antibody-based ELISA. Correlation with biologic activity. Allergy. 1997; 52: 806–813. 9284979 10.1111/j.1398-9995.1997.tb02151.x

[22] Fahlbusch B, Müller WD, Schlenvoigt G, Jäger L, Wahl R, Weber B. Monoclonal antibody immunoassay for quantitative analysis of group V allergens in grass pollen extracts. Clin Exp Allergy. 1993; 23: 747–754. doi: 10.1111/j.1365-2222.1993.tb00362.x 10779305 10.1111/j.1365-2222.1993.tb00362.x

[23] Meier U. Entwicklungsstadien mono- und dikotyler Pflanzen. Die erweiterte BBCH Monographie 2; 2001.

[24] Petrovska N, Wu X, Donato R, Wang Z, Ong EK, Jones E, et al Transgenic ryegrasses (Lolium spp.) with down-regulation of main pollen allergens. Mol Breeding. 2005; 14: 489–501. doi: 10.1007/s11032-005-1011-6

[25] Galán C, Smith M, Thibaudon M, Frenguelli G, Oteros J, Gehrig R, et al Pollen monitoring. Minimum requirements and reproducibility of analysis. Aerobiologia. 2014; 30: 385–395. doi: 10.1007/s10453-014-9335-5

[26] Albertine JM, Manning WJ, DaCosta M, Stinson KA, Muilenberg ML, Rogers CA. Projected carbon dioxide to increase grass pollen and allergen exposure despite higher ozone levels. PLoS One. 2014; 9: e111712 doi: 10.1371/journal.pone.0111712 25372614 10.1371/journal.pone.0111712 PMC4221106

[27] Suphioglu C, Singh MB, Simpson RJ, Ward LD, Knox RB. Identification of canary grass (Phalaris aquatica) pollen allergens by immunoblotting. IgE and IgG antibody-binding studies. Allergy. 1993; 48: 273–281. doi: 10.1111/j.1398-9995.1993.tb00728.x 7687099 10.1111/j.1398-9995.1993.tb00728.x

[28] Fahlbusch B, Hornung D, Heinrich J, Dahse HM, Jäger L. Quantification of group 5 grass pollen allergens in house dust. Clin Exp Allergy. 2000; 30: 1646–1652. doi: 10.1046/j.1365-2222.2000.00926.x 10.1046/j.1365-2222.2000.00926.x 11069575

[29] Chapman MD. Allergen specific monoclonal antibodies. New tools for the management of allergic disease. Allergy. 1988; 43: 7–14. doi: 10.1111/j.1398-9995.1988.tb05042.x 10.1111/j.1398-9995.1988.tb05042.x 3354797

[30] Flicker S, Vrtala S, Steinberger P, Vangelista L, Bufe A, Petersen A, et al A human monoclonal IgE antibody defines a highly allergenic fragment of the major timothy grass pollen allergen, Phl p 5: molecular, immunological, and structural characterization of the epitope-containing domain. J Immunol. 2000; 165: 3849–3859. 11034391 10.4049/jimmunol.165.7.3849

[31] Schappi GF, Monn C, Wuthrich B, Wanner HU. Direct determination of allergens in ambient aerosols: methodological aspects. Int Arch Allergy Immunol. 1996; 110: 364–370. doi: 10.1159/000237329 8768804 10.1159/000237329

[32] Fahlbusch B, Schlenvoigt G, Müller WD, Weber B, Jäger L. A two-site monoclonal antibody ELISA for the quantification of group V allergens in grass extracts. Clin Exp Allergy. 1994; 24: 752–757. 7982125 10.1111/j.1365-2222.1994.tb00986.x

[33] Klysner S, Welinder KG, Lowenstein H, Matthiesen F. Group V allergens in grass pollens: IV. Similarities in amino acid compositions and NH2-terminal sequences of the group V allergens from Lolium perenne, Poa pratensis and Dactylis glomerata. Clin Exp Allergy. 1992; 22: 491–497. 1611548 10.1111/j.1365-2222.1992.tb00152.x

[34] R Core Team. R: A Language and Environment for Statistical Computing. Vienna, Austria; 2015. Available: https://www.R-project.org/.

[35] Levin M, Rotthus S, Wendel S, Najafi N, Kallstrom E, Focke-Tejkl M, et al Multiple independent IgE epitopes on the highly allergenic grass pollen allergen Phl p 5. Clin Exp Allergy. 2014; 44: 1409–1419. doi: 10.1111/cea.12423 25262820 10.1111/cea.12423 PMC4278554

[36] Sharma A, Sharma N, Bhalla P, Singh M. Comparative and evolutionary analysis of grass pollen allergens using brachypodium distachyon as a Model System. PLoS One. 2017; 12: e0169686 doi: 10.1371/journal.pone.0169686 28103252 10.1371/journal.pone.0169686 PMC5245863

[37] Smith PM, Ong EK, Knox RB, Singh MB. Immunological relationships among group I and group V allergens from grass pollen. Molecular Immunology. 1994; 31: 491–498. doi: 10.1016/0161-5890(94)90068-X 7514270 10.1016/0161-5890(94)90068-x

[38] Chapman MD, Ferreira F, Villalba M, Cromwell O, Bryan D, Becker W, et al The European Union CREATE project: a model for international standardization of allergy diagnostics and vaccines. J Allergy Clin Immunol. 2008; 122: 882–889.e2. doi: 10.1016/j.jaci.2008.07.030 18762328 10.1016/j.jaci.2008.07.030

[39] Bundessortenamt Hannover., editor. Beschreibende Sortenliste 2013; 2013.

[40] Canova C, Heinrich J, Anto JM, Leynaert B, Smith M, Kuenzli N, et al The influence of sensitisation to pollens and moulds on seasonal variations in asthma attacks. Eur Respir J. 2013; 42: 935–945. doi: 10.1183/09031936.00097412 23471350 10.1183/09031936.00097412 PMC3787817

